# Biochemical and Functional Analysis of *Drosophila-Sciara* Chimeric Sex-Lethal Proteins

**DOI:** 10.1371/journal.pone.0065171

**Published:** 2013-06-10

**Authors:** María Fernanda Ruiz, Francesca Sarno, Silvia Zorrilla, Germán Rivas, Lucas Sánchez

**Affiliations:** 1 Centro de Investigaciones Biológicas, Consejo Superior de Investigaciones Científicas, Madrid, Spain; 2 Instituto de Química-Física “Rocasolano”, Consejo Superior de Investigaciones Científicas, Madrid, Spain; University of Maryland School of Medicine, United States of America

## Abstract

**Background:**

The *Drosophila* SXL protein controls sex determination and dosage compensation. It is a sex-specific factor controlling splicing of its own *Sxl* pre-mRNA (auto-regulation), *tra* pre-mRNA (sex determination) and *msl-2* pre-mRNA plus translation of *msl-*2 mRNA (dosage compensation). Outside the drosophilids, the same SXL protein has been found in both sexes so that, in the non-drosophilids, SXL does not appear to play the key discriminating role in sex determination and dosage compensation that it plays in *Drosophila*. Comparison of SXL proteins revealed that its spatial organisation is conserved, with the RNA-binding domains being highly conserved, whereas the N- and C-terminal domains showing significant variation. This manuscript focuses on the evolution of the SXL protein itself and not on regulation of its expression.

**Methodology:**

*Drosophila-Sciara* chimeric SXL proteins were produced. *Sciara* SXL represents the non-sex-specific function of ancient SXL in the non-drosophilids from which presumably *Drosophila* SXL evolved. Two questions were addressed. Did the *Drosophila* SXL protein have affected their functions when their N- and C-terminal domains were replaced by the corresponding ones of *Sciara*? Did the *Sciara* SXL protein acquire *Drosophila* sex-specific functions when the *Drosophila* N- and C-terminal domains replaced those of *Sciara*? The chimeric SXL proteins were analysed *in vitro* to study their binding affinity and cooperative properties, and *in vivo* to analyse their effect on sex determination and dosage compensation by producing *Drosophila* flies that were transgenic for the chimeric SXL proteins.

**Conclusions:**

The sex-specific properties of extant *Drosophila* SXL protein depend on its global structure rather than on a specific domain. This implies that the modifications, mainly in the N- and C-terminal domains, that occurred in the SXL protein during its evolution within the drosophilid lineage represent co-evolutionary changes that determine the appropriate folding of SXL to carry out its sex-specific functions.

## Introduction

**Figure 1:**
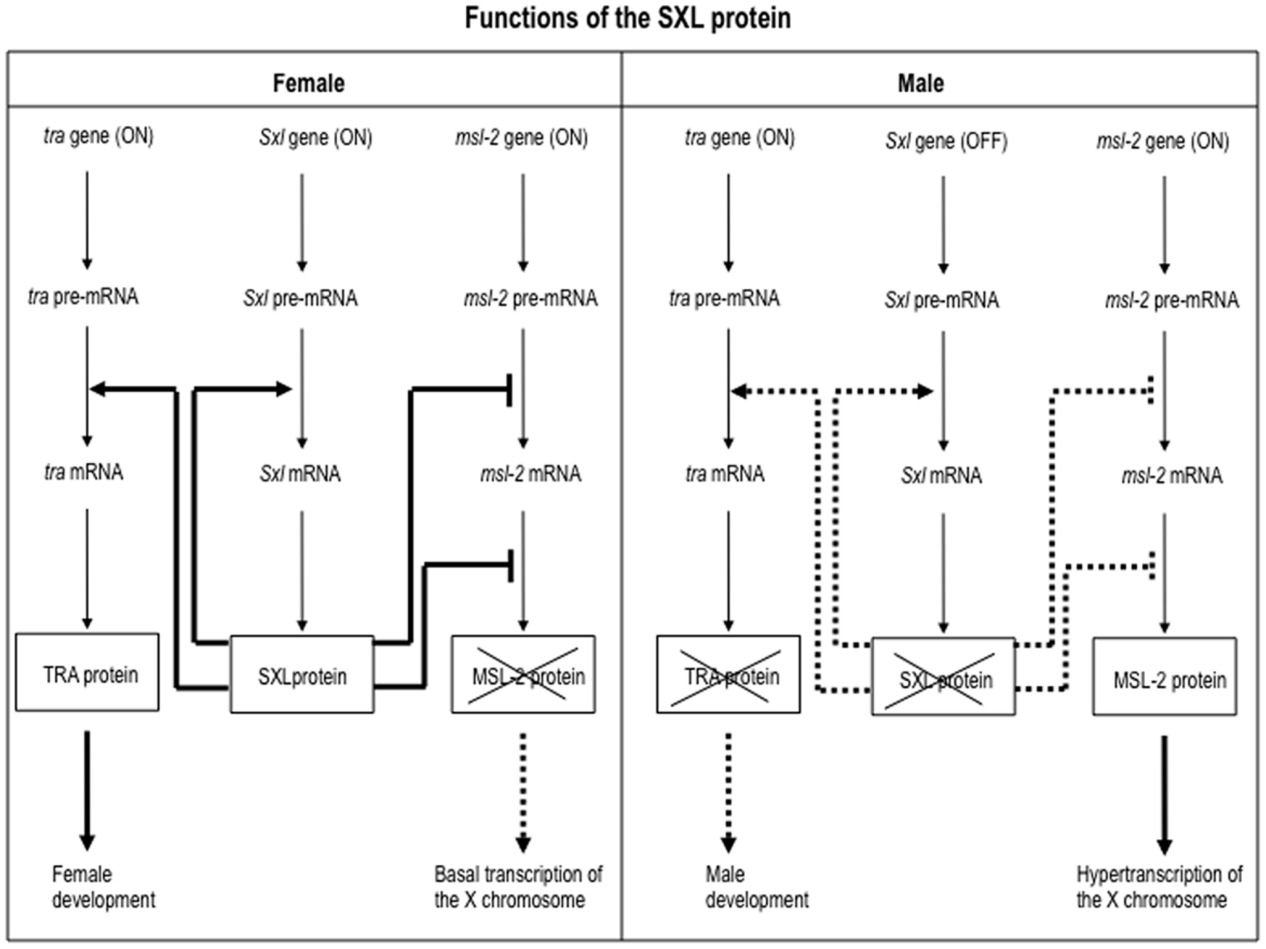
Scheme showing the sex-specific functions of the *Drosophila* SXL protein. Normal and dashed lines indicate active and inactive interactions, respectively. The crossed boxes for SXL, TRA and MSL-2 proteins designate lack of these proteins. After blastoderm stage, *Sxl* begins to function in both sexes, and production of the *Sxl* transcripts persist throughout the remainder of development and adult life. The male-specific transcripts are similar to their female-specific counterparts, except for the presence of an additional exon (exon 3), which contains translational stop codons. Consequently, male transcripts give rise to presumably inactive truncated proteins. In females, this exon 3 is spliced out and functional SXL protein is produced [67], [68]. The gene *tra* is transcribed in both sexes but its pre-mRNA follows an alternative splicing. In males, exon 2 introduces a translational stop codon, leading to the production of a truncated, presumably non-functional TRA protein. In females, however, approximately half of the *tra* pre-mRNA is spliced differently due to the intervention of the SXL protein, so that the RNA fragment on exon 2 containing the translation stop codon is not incorporated into the mature mRNA encoding the whole, functional TRA protein [6]–[10]. The gene *msl-2* is transcribed in both sexes but its pre-mRNA follows an alternative splicing. In females, the SXL protein prevents the splicing of an exon at the 5′ UTR, which introduces SXL-binding sequences [13]–[16]. Consequently, SXL binds to these sequences and to those located at the 3′UTR inhibiting the translation of the *msl2-* mRNA and then MSL2 protein is not synthesised [69], [70]. In males, however, the exon at the 5′ UTR is spliced out and MSL2 protein is produced.

In *Drosophila melanogaster*, the gene *Sex-lethal (Sxl)* controls both sex determination and dosage compensation (reviewed in [1]) (see Figure 1). The functional state of *Sxl* becomes fixed at blastoderm stage so that *Sxl* is activated in females but not in males [2], [3]. The capacity of *Sxl* to maintain its functional state throughout development and during the adult life of females is owed to its auto-regulatory function [4], manifested by the requirement of the SXL protein for the female-specific splicing of its own primary transcript [5]. SXL controls sex determination by regulating the female-specific splicing of the primary transcript from gene *transformer* (*tra*), so that only in females functional TRA protein is produced [6]–[10]. In *D. melanogaster*, dosage compensation is achieved in males by hyper-transcription of the single X chromosome and is controlled by the *msl’s* genes, whose products form the MSL complex that binds to the X chromosome (reviewed in [11], [12]). MSL is only formed in males because the presence of SXL protein in females prevents the production of protein MSL2 and consequently the formation of MSL. Thus, SXL controls dosage compensation by regulating the expression of gene *msl2*. This regulation takes place at the splicing and translational levels [13]–[16]. *Sxl* is also involved in the sexual development of the germ line (reviewed in [17]), yet the work presented here is focused on the soma and not on the germ line.

The SXL protein is a member of the RNA binding family of proteins. The analyses *in vitro* and *in vivo* of different *Drosophila* SXL-truncated protein constructs have determined that *Drosophila* SXL contains three well-defined domains: the central region formed by two RNA-binding domains, RBD1 and RBD2 (separated by a linker region), which endow to SXL with the capacity to bind to target sequences present in the *Sxl*, *tra* and *msl-2* pre-mRNAs; the amino-terminal domain that is involved in co-operation; and the carboxyl-terminal domain to which no specific function has been assigned, although it has been suggested that this domain might give structural stability to the protein [18]. Notwithstanding, conflicting results have been reported regarding the contribution of the SXL domains required for protein-protein interaction and, consequently, the co-operative binding of SXL. It has been claimed that the N-terminal region of SXL protein is involved in protein-protein interactions (SXL multimerisation) and is absolutely required for proper control of *Sxl* pre-mRNA alternative splicing [19]–[21]. According to Samuels *et al*. [22], however, protein-protein interaction is mediated by the RBDs domains, not by the amino terminal region, and can occur in the absence of additional, exogenous RNA. Sakashita and Sakamoto [23] have also reached the same conclusion concerning the importance of RBDs for SXL-SXL interaction but, in contrast to Samuels et al. [24] and in agreement with Wang and Bell [20], have claimed that homo-dimerization of SXL is RNA dependent. There is also some controversy concerning the function of the N-terminal domain of SXL in *tra* pre-mRNA sex-specific splicing regulation. It has been proposed that this region is not necessary for *tra* pre-mRNA splicing regulation [25], while others have proposed the opposite [10]. With respect to the control of dosage compensation by SXL protein, it has been reported that the N-terminal domain is not required for preventing *msl-2* expression [10], [26]. The two SXL RBD domains by themselves are able to control *in vitro msl-2* mRNA translation [26]. These contradictory results might be due to the different methodologies as well as the different SXL protein constructs used by the authors.

Deletions of the amino and the carboxyl termini do not interfere with the ability of SXL RBDs to properly bind *in vitro* to their target sequences. Nevertheless, both RNA binding domains in *cis* are required for site-specific RNA binding [20], [21], [24], [27]. The properties of several SXL protein constructs have been tested *in vitro* for their binding capacity [24]. Either RBD1 or RBD2 alone show reduced RNA binding activities. Duplications of the RBDs (RBD1-RBD1 and RBD2-RBD2) do not affect the RNA binding capacity but interfere with RNA recognition properties. Proteins in which the order of the two RBDs has been reversed (RBD2-RBD1) bind very weakly to oligonucleotides that contain only a single SXL-binding site. Nevertheless, the binding is close to normal if an oligonucleotide containing two binding sites is used as a probe, reflecting possible reestablishment of protein-protein interactions.

The *Sxl* gene has been characterised in different *Drosophila* species, *D. virilis* [28] and *D. subobscura* [29]. As in *D. melanogaster, Sxl* regulation occurs by female-specific alternative splicing. Outside the genus *Drosophila*, *Sxl* has been characterised in the dipterans *Chrysomya rufifacies* (blowfly) [30], *Megaselia scalaris* (the phorid fly) [31], [32] and *Musca domestica* (the housefly) [33], in the tephritids *Ceratitis capitata* (Medfly) [34] and *Bactrocera oleae* (olive fly) [35] (all of which belong to the suborder Brachycera), and in *Sciara ocellaris* [36], *Sciara coprophila, Rynchosciara americana* and *Trichosia pubescens* [37], which belong to the suborder Nematocera. *Sxl* has been also characterised in the lepidopteron *Bombyx mori* [38]. The *Sxl* gene of all these species is not regulated in a sex-specific manner, and therefore the same *Sxl* transcript encoding the functional SXL protein is found in both males and females. Thus, in the non-drosophilids, *Sxl* does not appear to play the key discriminating role in sex determination that it plays in *Drosophila*, but it seems to have a non-sex-specific function. Furthermore, in *Sciara*, where males are X0;2A and females are 2X;2A (reviewed in [39], [40]) and dosage compensation appears to be achieved by hypertranscription of the single X chromosome in males [41] −although different proteins seem to implement dosage compensation in *Drosophila* and *Sciara* [42]− the SXL protein has been found in polytene chromosomal regions of all actively transcribing chromosomes, co-localising with RNA polymerase II −as expected for a general splicing factor− but not with RNA polymerase I. This has been observed in both sexes in *S. ocellaris* [36], and in *S. coprophila, R. americana* and *T. pubescens* [37]. These results agree with the proposition that the non-drosophilist SXL protein might be involved in general non-sex specific gene regulation at the splicing and/or translational levels that would correspond to its ancestral non-sex specific function.

This manuscript focuses on the evolution of the SXL protein itself and not on the regulation of its expression. It is common that the arising of proteins with new functions being preceded by duplication of the gene encoding the original protein, followed by modification of one of the duplicated copies. The SXL protein is an example [43], [44]. The question naturally arises regarding which of the features present in the extant *Drosophila* protein have profited from the ancestral SXL protein and which ones have evolved during the phylogenetic lineage that gave rise to the drosophilids. The work here presented tries to address this question. To this respect, chimeric proteins between the SXL proteins of *D. melanogaster* and *S. ocellaris* were generated and their function tested on *D. melanogaster* sex determination and dosage compensation.

## Results

### Binding of the *Drosophila-Sciara* Chimeric SXL Proteins to *Drosophila* SXL-Binding Sites

The binding strength of both *Drosophila* (RBDs-mel) and *Sciara* (RBDs-sci) RNA-binding domains without the N- and C-terminal domains to SXL-binding poly(U) sequences was similar: K_d_ for *Drosophila*-RBDs was 350±50 µM and K_d_ for *Sciara*-RBDs was 340±40 µM (± refers to 95% confidence interval; t-test: P value = 0,27; P>0,5). Four mel-sci chimeric SXL proteins were then constructed by interchanging the N- and C-terminal domains of *Drosophila* and *Sciara* SXL proteins: chimera SX17 corresponds to the *Drosophila* SXL protein with the N-terminal domain of *Sciara* SXL; chimera SX64 corresponds to the *Drosophila* SXL protein with the C-terminal domain of *Sciara* SXL; chimera SX35 corresponds to the *Sciara* SXL protein with the N-terminal domain of *Drosophila* SXL and chimera SX28 corresponds to the *Sciara* SXL protein with the C-terminal domain of *Drosophila* SXL. As control, we used the normal *Drosophila* (SXM) and *Sciara* (SXS) SXL proteins. In all cases, they corresponded to full-length proteins.

The binding capacity of these chimeric proteins (GST-SXL fusion constructs) to SXL-binding poly(U) sequences was checked by *in vitro* RNA-binding assays (EMSA) using as substrate an RNA fragment containing a single copy of the poly(U) sequence located upstream and adjacent to the male-specific exon of *Drosophila Sxl*. Three replicas for each SXL protein were performed (for details see Material and Methods). The binding of the GST-SXL fusion proteins was due to SXL and not to GST since this by itself did not show binding to the poly(U)sequence, and secondly the binding was specific as the GST-SXL proteins did not bind to a non-poly(U) sequence (data not shown). The results are presented in Table 1. The *Drosophila* SXL protein (SXM) showed a binding ability significantly higher than that of *Sciara* (SXS) (t-test: P value = 8,216; 0,002<P<0,001). The binding capacity of the *Drosophila* SXL protein decreased when that of *Sciara* replaces its N-terminal domain (chimera SX17) (t-test: P value = 6,432; 0,005<P<0,002) or its C-terminal domain (chimera SX64) though in this latter case it was not significant (t-test: P value = 1,829; 0,2<P<0,1) (but see below). The binding capacity of the *Sciara* SXL protein improved when that of *Drosophila* replaces either its N-terminal domain (chimera SX35) (t-test: P value = 5,284; 0,01<P<0,005) or its C-terminal domain (chimera SX28) (t-test: P value = 5,952; 0,005<P<0,002) although this improvement did not reach the capacity shown by the own *Drosophila* SXL protein (SXM).

**Table 1:** Binding of the chimeric SXL proteins to *Drosophila* SXL-binding sequences.

SXL protein	N-terminal domain	RBD domains	C-terminal domain	K_d_ (µM) for single SXL-binding site	K_d_ (µM) for double SXL-binding site
**SXM**	*Drosophila*	*Drosophila*	*Drosophila*	150±20	0,5±0,3
**SXS**	*Sciara*	*Sciara*	*Sciara*	450±60	0,3±0,2
**SX17**	*Sciara*	*Drosophila*	*Drosophila*	350±50	2,4±0,5
**SX64**	*Drosophila*	*Drosophila*	*Sciara*	180±20	2,3±0,4
**SX35**	*Drosophila*	*Sciara*	*Sciara*	230±40	0,9±0,4
**SX28**	*Sciara*	*Sciara*	*Drosophila*	230±50	3,8±0,5

It is indicated the origin of the different domains that compose the SXL proteins. “Drosophila” stands for *Drosophila melanogaster* y “Sciara” stands for *Sciara ocellaris*. ± refers to 95% confidence interval.

**Figure 2:**
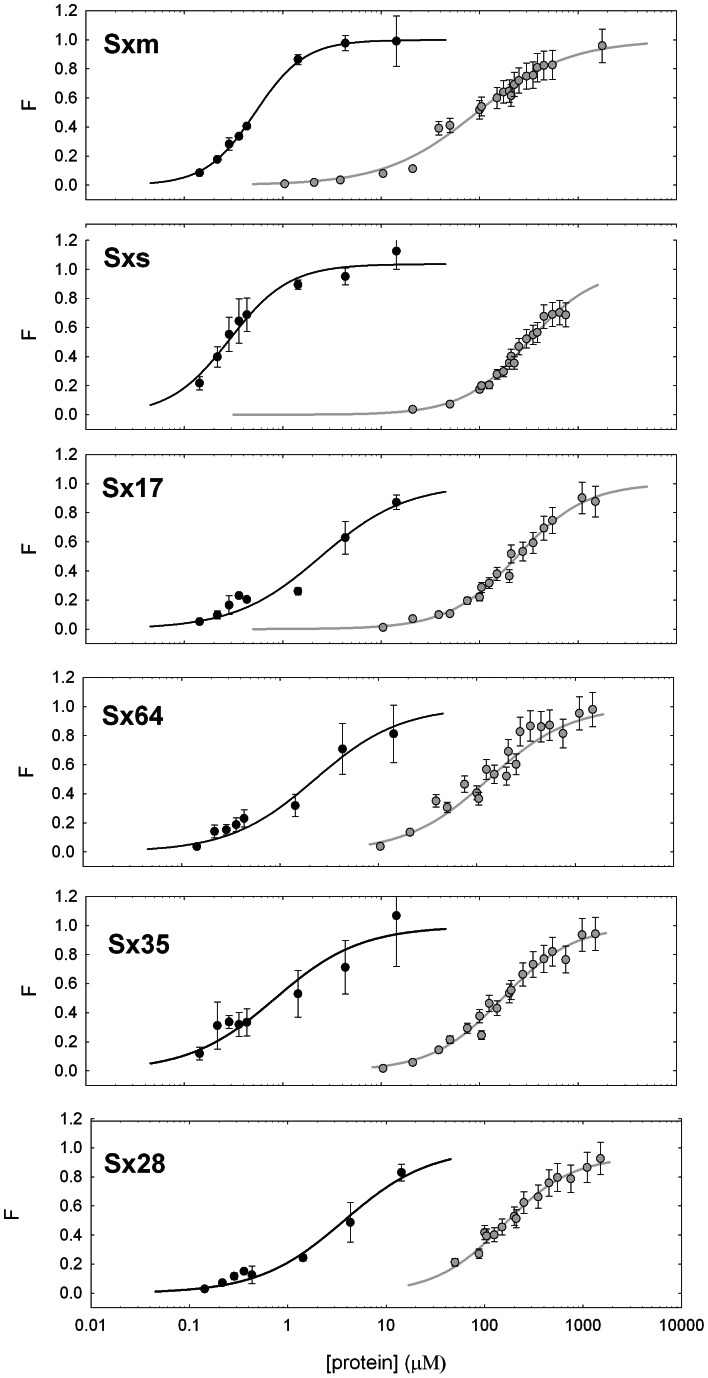
Biochemical characterization of SXL proteins. Quantitative analysis of the EMSA’s (an example is shown in Figure S1) for studying the properties of the SXL proteins to bind to RNA ligands carrying either single (grey) or double (black) poly(U) sequences. The fraction of bound RNA was quantified and plotted as a function of SXL protein concentration. Solid lines correspond to the best fit of Hill eqn.1 to the binding data obtained from titration of RNAs, with the best-fit parameters written in Table 1. “F” is defined by Eqn. 1, which is described in Materials and Methods.

The N-terminal domain of *Drosophila* SXL protein is involved in the co-operative binding of SXL to RNAs containing two or more poly(U) sequences [20], [27]. To test the co-operative capacity of the chimeric SXL proteins, RNA-binding analyses were performed by using as substrate an RNA fragment containing two poly(U) sequences located in intron 2 of *Drosophila Sxl* pre-mRNA, which have been shown to bind SXL in a co-operative manner [20]. Three replicas for each SXL protein were performed (for details see Material and Methods). The results are shown in Table 1 and Figure 2. The binding capacity of the normal *Drosophila* and *Sciara* SXL proteins, as well as of all chimeric proteins, significantly increased, as expected by the presence of two RNA target sequences in tandem. No significant differences were observed in this scenario for the normal *Drosophila* and *Sciara* SXL proteins (t-test: P value = 0,966; 0,5>P>0,2), but a significant reduction was observed for the *Drosophila* SXL protein (SXM) when its amino terminal (chimera SX17) (t-test: P value = 5,654; 0,005<P<0,002) or its carboxyl terminal (chimera SX64) (t-test: P value = 6,25; 0,005<P<0,002) regions were replaced by the corresponding ones of *Sciara*. A reduction was also observed for the *Sciara* SXL protein (SXS) when the amino (chimera SX35) (t-test: P value = 2,316; 0,05<P<0,1) or carboxyl (chimera SX28) (t-test: P value = 11,326; P<0,001) terminal domains were replaced by the corresponding *Drosophila* domains, with a highly significant binding reduction for SX28. Most importantly, whereas the binding of *Drosophila* SXL protein (SXM) was cooperative (Hill n>2), the binding of the *Sciara* SXL protein (SXS) and the four chimeric SXL proteins was best described with an independent binding site scheme, without co-operation (Hill n = 1).

Collectively, these results agree with the idea that both the binding strength and the co-operation of the normal and chimeric SXL proteins are not given by a specific domain of the protein but it depends on the whole protein.

The EMSA’s showed that the *Sciara* SXL protein formed aggregates that remained in the wells of the gel, not entering into the lane, in contrast to the behaviour shown by the *Drosophila* SXL protein. This latter protein, however, formed aggregates when its C-terminal domain was replaced by that of *Sciara* (chimera SX64), whereas the *Sciara* SXL protein lost this aggregation capacity when that of *Drosophila* replaced its C-terminal domain (chimera SX28) (Figure S1). This aggregation capacity was also observed for the chimera SX35 but not for the chimera SX17; the first carried the C-terminal domain of *Sciara* SXL whereas the second contained the C-terminal domain of *Drosophila* SXL. Thus, it seems that the C-terminal domain of *Sciara* SXL exhibits aggregation capacity. This feature was fundamentally dependent on the presence of poly(U) ligand since by itself the *Sciara* SXL protein did show a very low aggregation capacity (Figure S2). How much this aggregation property affects the binding capacity as well as the co-operative ability of the SXL proteins remains to be determined.

### Effect of the *Drosophila-Sciara* Chimeric SXL Proteins in *Drosophila*

To test the function *in vivo* of the *Drosophila-Sciara* chimeric and the normal SXL proteins of *D. melanogaster* and *S. ocellaris*, as control, the corresponding ORFs were linked to *UAS* sequences and transgenic *D. melanogaster* flies were generated. To express the transgenic SXL proteins, the *Arm-GAL4* driver line, which drives expression ubiquitously, was used. As expected, none of the transgenic *D. melanogaster* lines expressed the corresponding transgene in the absence of GAL4. If any basal expression existed, this would be irrelevant since XX and XY flies were normal, fertile females and males respectively. The effect of the transgenes was analysed by monitoring the viability of males since the expression of the SXL protein in males impairs the dosage compensation process what causes their lethality (see Introduction and Figure 1). Thus, the male-specific lethality was used as criterion for the *Sxl*-function of chimeric SXL proteins. In addition, since the *D. melanogaster* males do not express the full, female-specific functional SXL protein, this allowed us to test the direct effect of the SXL transgenes without interference of the wild type endogenous *Sxl* copy present in females (see below).

The four transgenic lines for the *Drosophila* SXL protein (*Sxm* transgene) caused full lethality to males. The six transgenic lines for the *Sciara* SXL protein (*Sxs* transgene) did not affect male viability. The four transgenic lines for the chimeric SX17 protein (*Sx17* transgene) caused full lethality to males. Among the five transgenic lines for the chimeric SX64 protein (*Sx64* transgene) four of them showed differently male lethality that ranges from 82 to 95%, whereas the remaining line did not affect male viability. The five transgenic lines for the chimeric SX35 protein (*Sx35* transgene) showed different effect on male lethality that ranges from 21 to 98%. Finally, among the four transgenic lines for the chimeric SX28 protein (*Sx28* transgene) only one line caused a minor male lethality (13%) whereas the rest of the lines did not affect male viability. The different effect of the same transgene is likely due to its different expression caused by the distinct chromosome location where the transgene was inserted. None of the transgenic lines affected females. Therefore, for functional analysis we selected the transgenic line showing the strongest male-specific lethality as the representative of each corresponding chimeric SXL protein.

The effect of the transgenes was analysed in males carrying the normal endogenous *Sxl* ^+^ allele on its X chromosome. It has been shown that transient expression of the *Drosophila* SXL protein in normal XY males causes the establishment of the auto-regulatory function of the endogenous *Sxl* ^+^ copy [5]. Therefore, the effect of the *Sxl* transgenes on male viability described above could be due to its effect on the endogenous *Sxl* ^+^ allele; that is, the transgenic SXL protein imposed to the endogenous *Sxl* ^+^ pre-mRNA the female-mode of splicing so that endogenous normal, functional SXL protein was produced resulting in the establishment of the auto-regulatory function of the endogenous *Sxl* ^+^ copy, and consequently in the permanent production of normal female SXL protein causing the male lethality. To circumvent this problem and to test the direct effect of the chimeric SXL proteins on male viability, these proteins were expressed in males carrying a null endogenous *Sxl* allele that does not produce functional SXL protein. The results of this analysis are presented in Table 2 (for details see Footnote to this Table).

**Table 2:** Effect of the transgenic *Drosophila-Sciara* chimeric SXL proteins on male viability with and without endogenous normal *Sxl* allele.

SXL protein	N-terminal domain	RNA-binding domains	C-terminal domain	Males with a wild type *Sxl^+^* endogenous copy		Males with a null *Sxl* endogenous copy	
				Control	Experimental (frequency)	Control	Experimental (frequency)
**SXM**	*Drosophila*	*Drosophila*	*Drosophila*	234	0 (0)	235	0 (0)
**SXS**	*Sciara*	*Sciara*	*Sciara*	196	211 (1,07)	219	214 (0,98)
**SX17**	*Sciara*	*Drosophila*	*Drosophila*	249	0 (0)	242	221 (0,91)
**SX64**	*Drosophila*	*Drosophila*	*Sciara*	220	12 (0,05)	173	69 (0,40)
**SX35**	*Drosophila*	*Sciara*	*Sciara*	217	5 (0,02)	298	39 (0,13)
**SX28**	*Sciara*	*Sciara*	*Drosophila*	245	213 (0,87)	163	172 (1,05)

The crosses to produce the males with a wild type endogenous *Sxl* allele are the following. For SXM, females *yw; Sxm/MKRS,Sb* & males *w/Y; arm-GAL4[w^+^]*; for SXS, females *yw; Sxs/MKRS,Sb* & males *w/Y; arm-GAL4[w^+^]*; for SX17, females *yw; Sx17/MKRS,Sb* & males *w/Y; arm-GAL4[w^+^]*; for SX64, females *yw; Sx64/CyO,Cy* & males *w/Y; arm-GAL4[w^+^]*; for SX35, females *yw; Sx35/CyO,Cy* & males *w/Y; arm-GAL4[w^+^]*; and for SX28, females *yw; Sx28/CyO,Cy* & males *w/Y; arm-GAL4[w^+^]*. Experimental males refer to those carrying the transgene and control to those carrying the balancer chromosome. The crosses to produce the males with an endogenous *Sxl* null allele are the following. The females in all crosses were *ywSxl^f1^ct^6^/FM7; arm-Gal4,w^+^* and the males were: for SXM, *yw/Y; Sxm/MKRS,Sb*; for SXS, *yw/Y; Sxs/MKRS,Sb*; for SX17, *yw/Y; Sx17/MKRS,Sb*; for SX64, *yw/Y; Sx64/CyO,Cy*; for SX35, *yw/Y; Sx35/CyO,Cy*; and for SX28, *yw/Y; Sx28/CyO,Cy*. Experimental males refer to those carrying the endogenous *Sxl* null allele plus the transgene and control to those carrying the endogenous *Sxl* null allele plus the balancer chromosome.

The *Drosophila* SXL protein (SXM) caused full lethality to males either with or without an endogenous *Sxl* ^+^ allele, as expected since this gene controls dosage compensation (see Figure 1). The replacement of its N- or C-terminal domains by those of *Sciara* appeared to impair male viability albeit with different degree that depends on the status of the endogenous *Sxl* copy. The substitution of the N-terminal domain (chimeric SX17 protein) produced full lethality that was practically suppressed (0,9% lethality) when the endogenous *Sxl* ^+^ allele was substituted by a null allele. This suggests that the male lethality is not a direct effect of this chimeric protein, which seems not to affect by itself dosage compensation, but an indirect effect through the endogenous *Sxl* ^+^ allele: the SX17 protein would set up the auto-regulation of endogenous *Sxl* ^+^ copy by imposing the female-specific splicing to its *Sxl* pre-mRNA. The replacement of the C-terminal domain (chimeric SX64 protein) produced a severe male lethality (95%) that was partially suppressed (60% lethality) when the endogenous *Sxl* ^+^ allele was substituted by a null allele. This suggests that this chimeric protein by itself can to a certain extent disturb male viability by damaging the dosage compensation process. The increase in lethality when the males contain an *Sxl* ^+^ allele further implies that SX64 seems to affect also *Sxl* pre-mRNA splicing regulation so that it is capable of establishing the auto-regulation of the endogenous *Sxl* ^+^ allele.

The *Sciara* SXL protein (SXS) and essentially the chimeric SX28 protein (the *Sciara* protein with the C-terminal domain of *Drosophila*) did not practically affect the viability of males either with or without an endogenous *Sxl* ^+^ allele. This suggests that these proteins are not capable of establishing the auto-regulatory function of the endogenous *Sxl* ^+^ copy and that by themselves do not seem to affect dosage compensation. Nevertheless, the replacement of its N- or C-terminal domains by those of *Drosophila* appeared to impair male viability albeit with different degree. The substitution of the N-terminal domain (chimeric SX35 protein) produced a severe male lethality (98%) that was slightly reduced (87% lethality) when the endogenous *Sxl* ^+^ allele was substituted by a null allele. This suggests that this chimeric protein by itself disturbs male viability via damaging the dosage compensation process, and furthermore it makes possible the auto-regulation of the endogenous *Sxl* ^+^ allele acting on the splicing regulation of its primary transcript.

In all the cases, the viability of transgenic females was not affected whether they carried one or two doses of the endogenous *Sxl* ^+^ allele (data not shown). Moreover, in all cases, the transgenic males that survived showed normal male external morphology. In addition, none of them showed the female- but the male-specific splicing of *tra* pre-mRNA (data not shown). This does not imply necessarily that the transgenic SXL proteins do not have a putative effect on sex determination. It could be attributed to the fact that because SXL controls dosage compensation, the males that survived are those in which the production of transgenic SXL protein was not sufficient to damage the dosage compensation process and then to affect their sexual development. To circumvent this problem, the *HS-GAL4* driver was used and the cultures were subjected to a daily heat-shock regime at 37°C for 1 hour throughout development. We were trying to see if the amount of produced chimeric SXL protein was insufficient to affect male viability but sufficient to impose the female sexual development. This protocol did not affect the viability of any of the transgenic males, which showed a normal male morphology (data not shown), suggesting that the heat-shock treatment did not induce sufficient amount of transgenic SXL protein to compromise both sex determination and dosage compensation.

**Figure 3:**
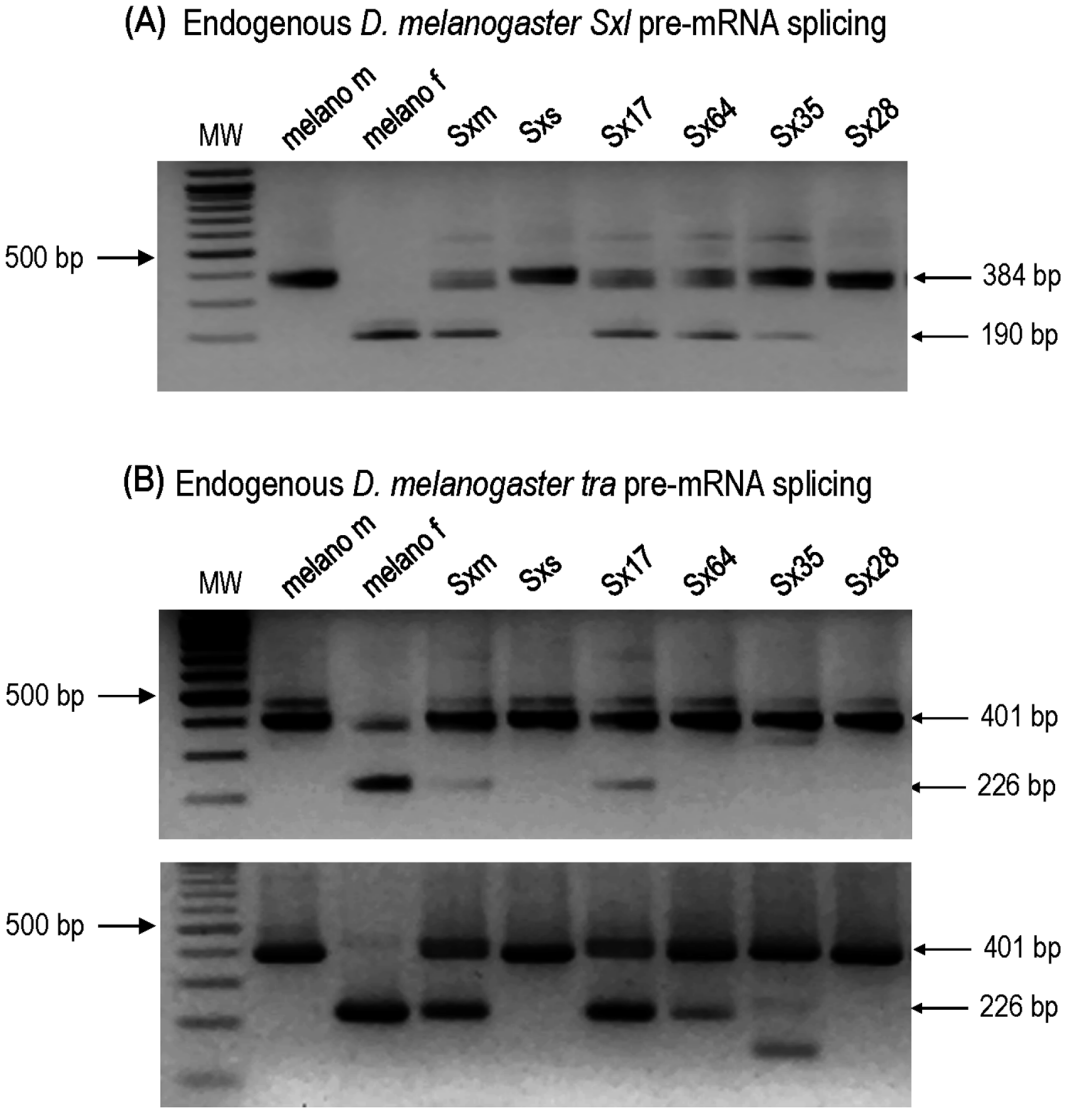
Effect of the SXL proteins on the sex-specific splicing of endogenous *Sxl* (A) and *tra* pre-mRNAS (B) in males carrying a null allele of *Sxl*. The conditions and primers for the RT-PCRs are described in Materials and Methods. The constitution of the SXL proteins is described in the Tables; and “melano m” and “melano f” stand for *Drosophila* wild type male and female, respectively. The genotypes of the males were: Sxm stands for males *ywSxl^f1^ct^6^/Y; arm-Gal4,w^+^/+; Sxm/Tub-Gal80^ts^*; Sxs stands for males *ywSxl^f1^ct^6^/Y; arm-Gal4,w^+^/+; Sxs/Tub-Gal80^ts^*; Sx17 stands for males *ywSxl^f1^ct^6^/Y; arm-Gal4,w^+^/+; Sx17/Tub-Gal80^ts^*; Sx64 stands for males *ywSxl^f1^ct^6^/Y; arm-Gal4,w^+^/Sx64; Tub-Gal80^ts^*/+; Sx35 stands for males *ywSxl^f1^ct^6^/Y; arm-Gal4,w^+^/Sx35; Tub-Gal80^ts^/+*; and Sx28 stands for males *ywSxl^f1^ct^6^/Y; arm-Gal4,w^+^/Sx28; Tub-Gal80^ts^/+*. These males were produced by crossing females *ywSxl^f1^ct^6^/Y; arm-Gal4,w^+^/CyO,Cy; Tub-Gal80^ts^/MKRS,Sb* with males *yw/Y; Sxm/MKRS,Sb*; males *yw/Y; Sxs/MKRS,Sb*; males *yw/Y; Sx17/MKRS,Sb*; males *yw/Y; Sx64/CyO,Cy*; males *yw/Y; Sx35/CyO,Cy*; and males *yw/Y; Sx28/CyO,Cy*.

To get around these difficulties, the function of the chimeric SXL proteins was directly tested on the splicing regulation of *Sxl, tra* and *msl-2* primary transcripts (see Figure 1). The rationale of these studies is to prevent lethality of the transgenic males so that they can reach the adulthood, and then to express the transgenic SXL proteins. For this purpose the *GAL4/GAL80* system was used. The GAL80 protein inhibits GAL4 protein function. GAL80 is temperature sensitive, with 18°C the most permissive temperature and 30°C the most restrictive [45]. XY males carrying a null *Sxl* allele, the corresponding transgenic SXL protein (*UAS::Sxl*-transgene), the *Tub-Gal4* driver and *Tub-Gal80* were produced by allowing them to develop at 18°C. This was possible because the GAL80 protein inhibited the function of the GAL4 protein so that no transgenic SXL protein was synthesised. The transgenic adult males were collected and transferred to 30°C during three days to allow the production of transgenic protein since GAL80 was not now functional and the GAL4 protein activated the *UAS::Sxl*-transgene. The direct effect of the transgenic SXL proteins was assured because the males carried a null *Sxl* allele that gives rise to primary transcript but no protein. The results are shown in Figure 3 (for details see Materials and Methods, and legend to this Figure).

As expected, the *Drosophila* SXL protein (SXM) induced the female-specific splicing of the endogenous *Sxl* pre-mRNA as revealed by the small band in lane SXM (Figure 3A). The same band appeared in lanes SX17 and SX64 indicating that the replacement in the *Drosophila* SXL protein of its N-terminal (chimera SX17) or C-terminal (chimera SX64) domains by those of *Sciara* did not affect the capacity of these chimeric proteins to impose the auto-regulatory function to the endogenous *Sxl* ^+^ copy. The band corresponding to SX64 was slightly less intense than that of SX17, which was similar to the own transgenic *Drosophila* SXL protein (SXM), in agreement with the full and non-fully lethality caused by SX17 and SX64, respectively, to *Sxl* ^+^/*Y* males (Table 2). On the contrary, the *Sciara* SXL protein (SXS) had no effect on the splicing of the endogenous *Sxl* pre-mRNA since the small band corresponding to the female-specific splicing did not appear (lane SXS), indicating that this protein cannot establish the auto-regulatory function of the endogenous *Sxl* ^+^ copy, in agreement with the viability of *Sxl* ^+^ */Y* males expressing SXS (Table 2). Nevertheless, this function was partially recovered if the N-terminal domain of *Drosophila* replaced that of *Sciara* (lane SX35) but not if the C-terminal domain was the substituted one (lane SX28). This is consistent with the partial recovery of males lacking the endogenous *Sxl* ^+^ copy and expressing the chimera SX35, and the minor effect of chimera SX28 on viability of *Sxl* ^+^/*Y* males. For all transgenes, PCR reactions with RNA samples were performed to guarantee there was no contamination with genomic DNA (negative controls of PCR reactions).

Figure 3B shows the results of transgenic SXL proteins on sex determination through their effect on the splicing regulation of endogenous *tra* pre-mRNA. Whereas the effect of the transgenic proteins on *Sxl* pre-mRNA splicing was already detected after the first PCR following the RT reaction, their effect on *tra* pre-mRNA splicing failed to detect any amplification corresponding to the female-specific mRNA isoform; only the band (401 bp) corresponding to the non-sex specific mRNA isoform was amplified (data not shown). It has been reported a delay in the effect of transgenic *Drosophila* SXL protein on the splicing pattern of *tra* pre-mRNA in males with an endogenous *Sxl^+^* copy, and even a failure to detect the female-spliced *tra* mRNA isoform when the expression of the normal SXL protein was transiently induced in males lacking the endogenous *Sxl^+^* copy [5]. Hence, we performed a second PCR (upper gel in Figure 3B). Only the lanes corresponding to the *Drosophila* protein (SXM) and its chimera with the N-terminal of *Sciara* (SX17) presented the band (226 bp) corresponding to the female-specific splicing of *tra* primary transcript. Since the males have only the non-sex-specific *tra* mRNA isoform, the detection of the induced female-spliced isoform by the transgenic SXL proteins could be hampered by the preferential amplification of the male-specific isoform, which is more abundant. We then perform a third PCR but this time the extension time was shortened to 7 seconds so as to favour the amplification of the female-specific (226 bp) against the non-sex-specific (401 bp) band. The results are shown in Figure 3B (lower gel). The similar intensity of the female band for SX17 and SXM suggests that the *Drosophila* SXL protein with the N-terminal domain of *Sciara* (chimera SX17) appeared to be as efficient as the own *Drosophila* SXL protein (SXM) in controlling the female-specific splicing of *tra* pre-mRNA. However, the *Drosophila* SXL protein with the C-terminal domain of *Sciara* (chimera SX64) was less efficient as indicated by the lower intensity of the 226 bp band. The normal *Sciara* SXL protein (SXS) and the *Sciara* SXL protein with the C-terminal domain of *Drosophila* (chimera SX28) had no effect on *tra* pre-mRNA splicing regulation, whereas the *Sciara* SXL protein with the N-terminal domain of *Drosophila* (chimera SX35) had a certain effect though very little as revealed by the lower intensity of the female band. For all transgenes, PCR reactions with RNA samples were performed to guarantee there was no contamination with genomic DNA (negative controls of PCR reactions).

The outcome of transgenic SXL proteins on dosage compensation was studied through their effect on the splicing regulation of endogenous *msl-2* pre-mRNA. All the transgenic SXL proteins, including the own *Drosophila* protein, failed to induce the female-specific splicing of *msl-2* primary transcript. Following the reasoning for analysing the *tra* pre-mRNA splicing, a second and a third PCRs were performed without any positive result, even for the normal *Drosophila* SXL protein (data not shown). Except for the *Sciara* (SXS) and its chimera with the C-terminal domain of *Drosophila* (SX28), these were unexpected results since the rest of the transgenic SXL proteins caused male-specific lethality, as previously shown. Particularly surprising was the negative result of the own *Drosophila* SXL protein (SXM). Nevertheless, it could be argued that the transgenic proteins did not affect *msl-2* pre-mRNA splicing regulation but the translation of the mature *msl-2* mRNA, since SXL controls dosage compensation by regulating *msl-*2 expression not only at the splicing but also at the translational level of *msl-2* mRNA. Hence, the presence of the MSL2 protein was analysed. The results of the Western-blot with total proteins extracts from the transgenic males probed with the affinity-purified antibody to *D. melanogaster* MSL-2 protein [14] showed that this was present in all transgenic males, at similar amounts to that found in wild type males (data not shown).

The cases where the transgenic *Sxl* genes showed no effect on *Sxl, tra* and *msl-2* cannot be attributed to a failure in their expression since RT-PCR assays of total RNA from the transgenic males demonstrated the expression of the transgenes. Furthermore, the Western-blot with total proteins extracts from the transgenic *Sxs*, *Sx28* and *Sx35* males probed with the affinity-purified antibody to *S. ocellaris* SXL protein [36] showed the presence of the transgenic proteins (Figure S3). The antibody does not recognise *Drosophila* SXL protein [36]. Notwithstanding, the occurrence of transgenic SXM, SX17 and SX64 proteins was verified by their effect on *Sxl* and *tra* pre-mRNA splicing regulation (Figure 3A,B).

## Discussion

It is common that the arising of proteins with new functions being preceded by duplication of the gene encoding the original protein, followed by modification of one of the duplicated copies that acquires a new function (neo-functionalisation). The SXL protein that controls sex determination and dosage compensation in *Drosophila* is an example [43], [44]. The duplication event that gave rise to *Sxl* and its paralog *sister-of-Sex-lethal* (*ssx*) occurred in the Brachycera (to which *Drosophila* belongs) after the Nematocera (to which *Sciara* belongs) branched off and before the *Drosophila* species split [43]. It seems, however, that the molecular evolution of *Sxl* and *ssx* did not follow the classical evolutionary pattern of duplication and posterior neo-functionalization, but a sub-functionalization model [44], [46].

The domains composing proteins can be defined from the structural, functional or evolutionary point of view (reviewed in [47]). The first refers to protein segments that behave as folding (structural) entities; the second stands for the activity given to the protein; and the third makes reference to the degree of evolutionary conservation. The analyses *in vitro* and *in vivo* of different *Drosophila* SXL-truncated protein constructs determined that *Drosophila* SXL contains three well-defined domains (see Introduction). The comparison of SXL proteins from different insects, belonging to different genera and families, has revealed that its spatial organisation is conserved, with the RBD domains in the central region, showing the highest degree of conservation, whereas the N- and C-terminal domains showing significant variation [33], [34], [37], [43]. This high degree of conservation of RBDs at the amino acid level is not reflected at the nucleotide level, indicating that the great majority of nucleotide changes are synonymous, and that purifying selection is acting on the RBD domains [37], [46]. These results led to the proposal that the changes experienced by the *Drosophila* SXL protein during its evolution might be mainly located in its terminal domains [33], [34], [37].

To address the question about the molecular evolution of *Drosophila* SXL protein, *Drosophila-Sciara* chimeric SXL proteins were synthesised by inter-changing their N- and C-terminal domains and their functionality in *Drosophila* were tested. It is reasonable to assume that *Sciara* SXL behaves as a general splicing factor, representing the non-sex-specific function of SXL protein in the non-drosophilids and so the function of the ancient SXL from which the extant *Drosophila* SXL protein evolved [36], [37] (see Introduction). However, the *Drosophila* SXL behaves a sex-specific splicing factor, having three functions: (1) auto-regulation, which is manifested by its requirement to regulate its expression during development and adult life through its involvement in the female-splicing regulation of its own primary transcript; (2) control of sex determination by regulating the female-specific splicing of *tra* pre-mRNA to produce functional TRA protein only in females; and (3) control of dosage compensation through splicing and translation regulation of *msl-2* pre-mRNA and mRNA, respectively. Two questions were addressed. Did the *Drosophila* SXL protein have affected their functions when their N- and C-terminal domains were replaced by the corresponding ones of *Sciara*? Alternatively, did the *Sciara* SXL protein acquire *Drosophila* SXL sex-specific functions when the N- and C-terminal domains of this replace those of *Sciara*? The criteria used to test the functionality of the *Drosophila-Sciara* chimeric SXL proteins were the following:

1. The auto-regulatory function was studied by comparing the specific lethality of males with and without an endogenous *Sxl^+^* copy, and by analysing in males the splicing of *Sxl* pre-mRNA from an endogenous *Sxl* null allele.
2. The sex-determination function could not be checked by monitoring the ability of chimeras to impose the female development to males carrying a null allele of *Sxl*. Notwithstanding, the sex-determination function was checked by monitoring the effect of the chimeras on the female-specific splicing of endogenous *tra* pre-mRNA in males carrying an endogenous *Sxl* null allele.
3. The dosage compensation function was examined by analysing the specific lethality of males without an endogenous *Sxl^+^* copy. The viability of females was never compromised by the expression of transgenic SXL proteins so that the male-specific lethality is a *bona fide* indicator of dosage compensation upset. The study of these chimeras on the splicing and translation of *msl-2* pre-mRNA and mature mRNA, respectively, failed to detect any effect. This was unexpected for those cases where male-specific lethality was observed, especially in the case of the own *Drosophila* SXL protein. We have no reason for this result except to say that because this analysis was done on adult males, dosage compensation might not be so critical for the function of the adult somatic tissues as it is during development. It has been shown that dosage compensation exists in the germ line of *Drosophila* adults [48], [49], [50], although it has been claimed the opposite [51], but it appears that genes different from the *msl* genes implement this dosage compensation [52], [53], [54].

Since the *Drosophila* SXL protein exerts its functions through its capacity to bind to RNA, the biophysical properties of the normal *Drosophila* and *Sciara* SXL proteins as well as their chimeras were firstly tested. To this respect, their binding capacity to single and double poly(U) sites of *Drosophila* were studied. The results on this *in *vitro analysis are summarised. Firstly, although the binding capacity of both *Drosophila* and *Sciara* RNA-binding domains, without the N- and C-terminal domains, was similar, the whole SXL protein of *Drosophila* showed a binding ability higher than that of *Sciara* in the case of a single binding site, but both proteins presented a similar affinity in the case of a double binding-site. Secondly, the binding capacity of the *Drosophila* SXL protein decreased when that of *Sciara* replaces either its amino- or its carboxyl-terminal domain, whereas the binding capacity of the *Sciara* SXL protein improved when that of *Drosophila* replaces either its amino- or its carboxyl-terminal domain, although this improvement did not reach the capacity shown by the own *Drosophila* SXL protein. Finally, whereas the SXL protein of *Drosophila* showed co-operative properties, that of *Sciara* did not. Moreover, the co-operation exhibited by *Drosophila* SXL protein is impaired when either its N- or C-terminal domains were replaced by the corresponding ones of *Sciara* SXL protein. Similarly, this protein did not acquire co-operative properties when its N- or C-terminal domains were replaced by those of *Drosophila* SXL protein. Collectively, these results indicate that the binding capacity of SXL and its co-operative ability is a property of the whole protein rather than due to a specific domain.

The results of *in vivo* analyses regarding the function of the chimeric SXL proteins are summarised in Table 3. The normal *Sciara* SXL protein (SXS) and that carrying the C-terminal domain of *Drosophila* SXL (chimera SX28) did not show any of the functions of *Drosophila* SXL protein. The other three chimeric proteins presented different degrees of the auto-regulatory, the sex determination and the dosage compensation functions. It has been described that the N-terminal domain of *Drosophila* SXL protein is involved in protein-protein interactions (SXL multimerisation) and endows to SXL with co-operative function, which is absolutely required for proper control of *Sxl* pre-mRNA alternative splicing [18]–[21]. The *Drosophila* SXL protein showed auto-regulatory function when its N-terminal domain was replaced by the corresponding one of *Sciara* (chimera SX17), and a lower auto-regulatory function if the C-terminal domain is the one that was replaced (chimera SX64). The *Sciara* SXL protein gained some auto-regulatory function when the corresponding ones of *Drosophila* replaced its N-terminal (chimera SX35) but not its C-terminal (chimera SX28) domain. In addition these chimeras had no co-operative properties. These results suggest that co-operation *per se* is not absolutely necessary for *Sxl* auto-regulation but it matters the large-scale structure of the SXL protein, with the N-terminal domain playing a leading role. In this context, it is worth mentioning that the early SXL protein shows auto-regulatory function although the beginning of its N-terminus domain differs in amino acid sequence with respect to the sequence in the late SXL protein [55]. With respect to the sex determination function, the chimeras paralleled their behaviour on *Sxl* auto-regulation.

**Table 3:** Function of the transgenic *Drosophila-Sciara* chimeric SXL proteins.

SXL protein	N-terminal domain	RNA-binding domains	C-terminal domain	Auto-regulatory function	Sex determination function	Dosage compensation function
**SXM**	*Drosophila*	*Drosophila*	*Drosophila*	YES (+++)	YES (+++)	YES (+++)
**SXS**	*Sciara*	*Sciara*	*Sciara*	NO	NO	NO
**SX17**	*Sciara*	*Drosophila*	*Drosophila*	YES (+++)	YES (+++)	NO
**SX64**	*Drosophila*	*Drosophila*	*Sciara*	YES (++)	YES (++)	YES (+)
**SX35**	*Drosophila*	*Sciara*	*Sciara*	YES (+)	YES (+)	YES (++)
**SX28**	*Sciara*	*Sciara*	*Drosophila*	NO	NO	NO

The degree of functionality of SXL proteins is qualitatively indicated by the number of “+”.

The *Drosophila* SXL protein lost its dosage compensation function when its entire N-terminal region was replaced by the complete one of *Sciara* (chimera SX17). This result seems to be in contradiction with the reported result that the N-terminal domain of *Drosophila* SXL protein is not required for preventing *msl-2* expression [10], [26] and that the two SXL RBD domains by themselves are able to control *in vitro msl-2* mRNA translation [26]. This discrepancy might be explained by the different SXL protein constructs used. Whereas in this work a complete *Drosophila-Sciara* chimeric protein was employed, Gebauer et al. [26] used a truncated *Drosophila* SXL protein lacking the first 93 and the last 32 amino acids of the N- and C-terminal domains, respectively; and Yanowitz et al. [10] used a truncated *Drosophila* SXL protein lacking the first 38 amino acids of the N-terminal domain. Hence, the truncated *Drosophila* constructs and the whole *Drosophila-Sciara* chimeric protein are likely to have affected their global structure in a different way, what might determine their dissimilar function on dosage compensation. To this respect, the *Sciara* SXL protein gained some dosage compensation function when its N-terminal (chimera SX35) but not its C-terminal (chimera SX28) domain was replaced by the corresponding ones of *Drosophila*.

In general terms, there is agreement between the *in vitro* and the *in vivo* results, although the *in vitro* results cannot be straightforward extrapolated to the function of SXL *in vivo*. The results generated by *in vitro* analysis gave us information about the affinity and co-operation of SXL proteins in a scenario where the protein and the ligand (RNA sequence) were the only actors, whereas in the *in vivo* scenario other factors modulating the physical properties of the SXL and then its function came into play. Actually, it has been shown that the *Drosophila* SXL protein requires its interaction with other proteins encoded by the genes *snf* [56], [57], *fl(2)d* [58], [59] and *vir* [60], [61] to exert its function. Nevertheless, all the results presented here led us to propose that the functional properties of the extant *Drosophila* SXL protein depend on its global structure rather than on a specific domain; that is, the binding capacity of SXL, which is exerted through its two RNA binding domains, and SXL multimerisation, which seems to be implemented by the N-terminal domain, require the carboxyl-terminal domain. Furthermore, it is proposed here that the RNA-binding capacity of the *Drosophila* SXL protein might be a property already present in the ancestral SXL protein of the insects from which the dipterans evolved and that the modifications, mainly in the N- and C-terminal domains, that occurred in the SXL protein during its evolution within the drosophilid lineage represented co-evolutionary changes that determine the appropriate folding of SXL to carry out its sex-specific functions.

This assertion receives further support from the results regarding the effect of *ssx* null mutations on *Drosophila*: the lack of *ssx* function does affect neither the viability nor the sexual development of both males and females; that is, *ssx* does not have the sex-specific functions shown by *Sxl* [44]. Moreover, the comparison of SSX with *Drosophila* SXL ([43] and our own data) and *Sciara* SXL (our own data) revealed that the RBDs domains are also the best conserved (79% and 70% for *Drosophila* and *Sciara*, respectively), followed by the C-terminal domain (45% and 40% for *Drosophila* and *Sciara*, respectively), whereas the N-terminal domain showed very low similarity (8% and 5% for *Drosophila* and *Sciara*, respectively).

## Materials and Methods

### Flies and Crosses

*Drosophila* flies were cultured on standard food. For the description of the mutant alleles and GAL4 constructs see Lindsley and Zimm [62] and FlyBase.

### Molecular Analyses

Total RNA extracts from frozen adults were prepared using the Trizol-reagent kit (Invitrogen) following the manufacturer’s instructions. Five micrograms of total RNA from each sample were reversed transcribed with Superscript III (Invitrogen) following the manufacturer’s instructions. Reverse transcription reactions were performed with primer *Sxlmel6* (5′CCAGCGACAATCCGCAGAG3′) located in exon 5 of *D. melanogaster Sxl* for splicing analysis of the endogenous *Drosophila Sxl* gene; with primer *tramel2* (5′TGCTGCGACTTCGGCTATG3′) located in exon 2 of *D. melanogaster tra* gene for splicing analysis of the endogenous *Drosophila tra* gene, and with primer *msl2mel3* (5′GTCACCTTCAAGTATGCCGTC3′) located in exon 1 of *D. melanogaster msl-2* gene for splicing analysis of the *Drosophila msl-2* gene. Two percent of the synthesised cDNA was amplified by PCR. For splicing analysis of the endogenous *Drosophila Sxl* pre-mRNA, the primers used in the PCR were *Sxlmel5* (5′ACCGAAACTCACCTTCGATC3′) located in exon2 and primer *Sxlmel3* (5′CCGGATGGCAGAGAATGGGAC3′) located in exon 4. The expected size of the amplicon is 384 bp for male and 190 bp for female. For splicing analysis of the *Drosophila tra* pre-mRNA, the primers used in the PCR were *tramel1* (5′CAAGGTGCAAGCCGAGTAC3′) located in 5′ UTR and primer *tramel5* (5′AACCTCGTCTGCAAAGTACGG3′) located in exon 2 upstream to *tramel2*. The expected size of the amplicon is 401 bp for male and 226 bp for female. For splicing analysis of the *Drosophila msl-2* pre-mRNA, the primers used in the PCR were *msl2mel1* (5′CACACTGGCTTCGCTCAGC3′) and primer *msl2mel2* (5′CAGCCCAAAAGTGAGACTCC3′) located in 5′ UTR flanking the sex-specifically spliced intron. The expected size of the amplicon is 256 bp for male and 389 bp for female. The amplicons were analysed by electrophoresis in 2% agarose gels. In all cases, PCR reactions with RNA samples were performed to guarantee there was no contamination with genomic DNA (negative controls of PCR reactions).

### Construction and Purification of SXL Proteins

Figure S4 shows the location of all the primers used in the construction of the chimeric SXL proteins.

The GST expression system was used to produce the SXL fusion proteins following Smith and Johnson [63] with minor changes. To generate the GST-SXS fusion, the whole ORF of *Sciara Sxl* was amplified from cDNA with primers *Gex1* (5′CGGGGATCCAATCAGAGTGAGTGTCG3′) and *Gex2* (5′GCAAAGCTTATTAGCTTTCATCTCAATA3′) containing a restriction site for BamHI and HindIII, respectively. The amplicon was cloned in p*GEMT-easy* (Promega) and sequenced. The DNA of the p*GEMT-easy* vector containing the *Sciara Sxl* ORF was cut with BamHI and HindIII and the fragment was ligated in frame into the p*Gex-B* vector using the T4 DNA ligase (Roche). The GST-SXM fusion construct was obtained from Dr. J. Valcárcel [8].

To generate the GST-SX17 fusion, the sequence of the *Sciara Sxl* ORF encoding the N-terminal domain of SXL was amplified from cDNA with primers *Nter1s* (5′CGGGATCCAATGTACAATAAGAATGGGTATC3′) and *Nter2s* (5′TCTAGAGCCAGCACAGCCAGTTAG3′) containing a restriction site for BamHI and XbaI, respectively. The fragment of the *Drosophila Sxl* ORF encoding the two RNA-binding domains plus the C-terminal domain was amplified with primers *Rbd1m* (5′TCTAGAACCAACCTGATTGTCAACTAC3′) and *Cter2m* (5′GCAAAGCTTTCAGATAAACTTTTTAGCATG3′) containing a restriction site for XbaI and HindIII, respectively. To generate the GST-SX64 fusion, the sequence of the *Sciara Sxl* ORF encoding the C-terminal domain of SXL was amplified from cDNA with primers *Cter1s* (5′CTCGAGGGCAAACAGAAAGCGACC3′) and *Cter2s* (5′GCAAAGCTTTCAATATGGACTTATGTTCTG3′) containing a restriction site for XhoI and HindIII, respectively. The fragment of the *Drosophila Sxl* ORF encoding the N-terminal domain plus the two RNA-binding domains was amplified with primers *Nter1m* (5′CGGGATCCTATGTACGGCAACAATAATCC3′) and *Rbd2m* (5′CTCGAGCTCAGCCAACCGGACG3′) containing a restriction site for BamHI and XhoI, respectively. To generate the GST-SX35 fusion, the sequence of the *Drosophila Sxl* ORF encoding the N-terminal fragment of SXL was amplified from cDNA with primers *Nter1m* (above) and *Nter2m* (5′TCTAGATGCCCGAGGATCGTTCATG3′) containing a restriction site for BamHI and XbaI, respectively. The fragment of the *Sciara Sxl* ORF encoding the two RNA-binding domains plus the C-terminal domain was amplified with primers *Rbd1s* (5′TCTAGAACCAATTTAATTGTTAACTATTTAC3′) and *Cter2s* (above) containing a restriction site for XbaI and HindIII, respectively. To generate the GST-SX28 fusion, the sequence of the *Drosophila Sxl* ORF encoding the C-terminal fragment of SXL was amplified from cDNA with primers *Cter1m* (5′CTCGAGGGCAAGGCGAAGGCGGC3′) and *Cter2m* (above) containing a restriction site for XhoI and HindIII, respectively. The fragment of the *Sciara Sxl* ORF encoding the N-terminal domain plus the two RNA-binding domains was amplified with primers *Nter1s* (above) and *Rbd2s* (5′CTCGAGTTCGGCAACGCGTACGC3′) containing a restriction site for BamHI and XhoI, respectively. All the amplicons were cloned in p*GEMT-easy* (Promega) and sequenced. The DNA of p*GEMT-easy* vectors was cut with the corresponding restriction enzymes and the fragments were ligated in frame into the p*Gex-B* vector using the T4 DNA ligase (Roche). To generate the GST-RBDs-mel fusion, the sequence of *Drosophila Sxl* ORF encoding the two RNA-binding domains plus the linker was amplified with primers *D1* (5′ATGAAGGATCCTCGGGCAAGCA3′) and *D2* (5′GGAACCAAGCTTATCTACGACATAAAG3′) containing a restriction site for BamHI and HindIII, respectively. To generate the GST-RBDs-sci fusion, the sequence of *Sciara Sxl* ORF encoding the two RNA-binding domains plus the linker was amplified with primers *S1* (5′CTGGGATCCCAGCGGCACC3′) and *S2* (5′TCATGTTCAAGCTTGAATTTTAAAATTG3′) containing a restriction site for BamHI and HindIII, respectively. The amplicons were cloned in p*GEMT-easy* (Promega) and sequenced. The DNA of the p*GEMT-easy* vectors was cut with BamHI and HindIII and the fragments were ligated in frame into the p*Gex-A* vector using the T4 DNA ligase (Roche). All the positive clones were sequenced to ascertain correct orientation.

### Construction of *UAS::*Chimeric-*Sxl*-cDNA Transgenes

For the construction of the transgenes, the whole ORF of the corresponding chimeric SXL proteins (SX17, SX64, SX35 and SX28) plus the *Drosophila* (SXM) and *Sciara* (SXS) SXL proteins were amplified using as template the GST-SXL fusion constructions described above. The primers used for the *Sxm* transgene were p*UASSxl1m* (5′GAAGATCTATATGTACGGCAACAATAATCC3′) and p*UASSxl2m* (5′GGGGTACCTTCAGATAAACTTTTTAGCATCG3′) containing the restriction sites for BglII and KpnI, respectively. The primers used for the *Sxs* transgene were p*UASSxl1s* (5′GAAGATCTAATGTACAATAAGAATGGGTATC3′) and p*UASSxl2s* (5′GGGGTACCCTCAATATGGACTTATGTTCTG3′) containing the restriction sites for BglII and KpnI, respectively. The primers used for the *Sx17* and *Sx28* transgenes were p*UASSxl1s* and p*UASSxl2m*, and for the *Sx35* and *Sx64* transgenes were p*UASSxl1m* and p*UASSxl2s*, described above. The amplicons were cloned in p*GEMT-easy* (Promega) and sequenced. The DNA of the p*GEMT-easy* vectors was cut with BglII and KpnI and the fragments were ligated into p*UAST* vector. The microinjections for generating the transgenic *D. melanogaster* lines were performed by Genetic Services (Sudbury, MA, USA). Standard genetic crosses determined the chromosomal location of the transgenes. To ascertain that each transgenic line was carrying the correct transgene, RT-PCR analysis was performed and the amplicons corresponding to the whole transgenes were cloned and sequenced.

### Preparation of RNA Substrates for Binding Assays

The poly(U) sequence 5′ACAUAUUUUUUUUCACAGC3′ located at the 5′ end of the male-specific exon 3 of *D. melanogaster* was used as a substrate for RNA-binding assays. The RNA ligand was prepared as follows. An oligonucleotide with that poly(U) sequence preceded by the T7 promoter sequence was synthesised. This was used in *in vitro* transcription using T7 RNA polymerase and the Fluorescin RNA labelling mix kit (Roche). The same procedure was used for preparing the RNA substrate in the RNA-binding assays for testing cooperative capacity of the chimeric SXL proteins, except that in this case the RNA ligand contained two contiguous poly(U) sequences (5′CATGATTAUUUUUUUUUAUUUUUUUUCGGTGA3′) located in intron 3 of *D. melanogaster Sxl* pre-mRNA and known to bind Sxl in a co-operative manner [20].

*In vitro* transcribed RNA was precipitated by adding 0,1 volume of NaAc 3 mM and 2.5 volumes of absolute ethanol, and resuspended in sterile RNase-free water. The concentration was measured in Nanodrop.

### RNA Binding Assays (Electrophoretic Mobility Shift Assay, EMSA)

The SXL proteins were mixed with yeast tRNA (3 µg) in the binding buffer (20 mM Hepes at pH 8.0, 0,1 M KCl, 0,5 mM EDTA, 1 mM DTT, 0,05% NP40 and 20% glycerol) during 5 minutes on ice. The RNA substrate (2,6 µg for one single- and 2 µg for double-binding sites) is then added and incubated at room temperature for 10 minutes. The samples were loaded and resolved on a 5% non-denaturating polyacrylamide gel (60∶1 acrylamide to bis-acrylamide) in 0,25× TBE. A 15 minutes pre-run at 50 Volts was performed before loading the samples and the run lasted 1 hour at 250 Volts. The gel was analysed by using UV light Gel-Doc.

The binding of SXL proteins to RNAs containing one or two poly(U) sequences as measured by EMSA assays was well described by the empirical Hill function [64]: *F = (C/C_50_)^n^/(1+ (C/C_50_)^ n^)* (eqn. 1), where *F* is the fraction of complex at each point in the binding titration, *C* is the protein concentration, C_50_ is the protein concentration at half binding saturation, and *n* is a Hill coefficient. In the absence of a detailed molecular binding mechanism, this analysis allows estimating an apparent value for the dissociation constant, K_d_ (K_d_ = 1/C_50_, for n = 1) and to compare the binding properties of the different protein variants used in this study. A Matlab model script was written for fitting this model to the binding data.

### Western Blots

Samples of total proteins from adult transgenic males were prepared by homogenisation in STE buffer (10 mM Tris, pH 8.0, 150 mM NaCl, 1 mM EDTA) containing protease inhibitors complete Mini, EDTA free kit (Roche). SDS-polyacrylamide gels (8% for MSL2 protein or 12% for SXL protein) [65] were blotted onto nitrocellulose [66], blocked with 5% BSA, 10% non-fat dried milk and 0.05% Tween-20 in PBS, and hybridised with anti-MSL2 (1∶2000) [14] or anti-SXL (1∶1000), a polyclonal antibody against the *S. ocellaris* SXL protein [36], overnight at 4°C. After washing in 0.05% Tween-20 in PBS (TPBS), filters was incubated with the secondary antibody (goat anti-rabbit IgG-HRP conjugated (1∶2000) (Santa Cruz Biotechnology) for 1 h at room temperature. Filters were washed in TPBS and developed with the ECL Western blotting analysis kit (Amersham Pharmacia Biotech).

## Supporting Information

Figure S1 Examples of binding of SXL proteins to one single SXL-binding site. The amount of SXL proteins and RNA substrate is indicated in µg above each lane.
(TIFF)

Figure S2 EMSA **(A)** and Western-blot **(B)** for the interaction between the *Sciara* SXL (SXS) protein and the *Drosophila* SXL-binding site. **(A)** The RNA sequence is described in Materials and Methods. Lanes 1, 2, 3, 7, 8 and 9 corresponded to 3 µg of SXS protein used in the reaction, whereas lanes 4, 5, 6, 10, 11 and 12 corresponded to 0,7 µg of SXS protein used in the reaction. The arrow in indicates the wells of the gel. **(B)** Western-blot to demonstrate to existence of SXS protein retained in the wells of the EMSA shown in (A). The material retained in the wells was extracted and used for the Western–blot. Lane 1 corresponds to the material retained in the wells of lanes 1, 2 and 3; lane 2 corresponds to the material retained in the wells of lanes 7, 8 and 9; lane 3 corresponds to the material retained in the wells of lanes 4, 5 and 6, and lane 4 corresponds to the material retained in the wells of lanes 10, 11 and 12. C stands for the SXS protein alone and used as control. The Western-blot was hybridised with the serum against the *Sciara* SXL protein [36].
(TIFF)

Figure S3 Western-blot hybridised with serum against the *Sciara* SXL protein [36] showing the expression of the transgenic SXL proteins. The antibody does not recognise *Drosophila* SXL protein [36]. See text for details.
(TIFF)

Figure S4 Scheme showing the *Drosophila-Sciara* chimeric SXL proteins, where the location of the primers used for their construction is indicated. The sequences of the primers and the added sequences for the restriction enzymes are described in Materials and Methods. N-mel, RBD-mel and C-mel stand, respectively, for the amino-terminal domain, the two RNA-binding domains and the carboxyl-terminal domain of *Drosophila* SXL. N-sci, RBD-sci and C-sci stand, respectively, for the amino-terminal domain, the two RNA-binding domains and the carboxyl-terminal domain of *Sciara* SXL.
(TIFF)
