# Supplementary figures and images for: Biochemical and Functional Analysis of Drosophila-Sciara Chimeric Sex-Lethal Proteins
Source: PLoS One. 2013 Jun 10;8(6):e65171. doi: 10.1371/journal.pone.0065171 (PMC3677924; doi:10.1371/journal.pone.0065171)

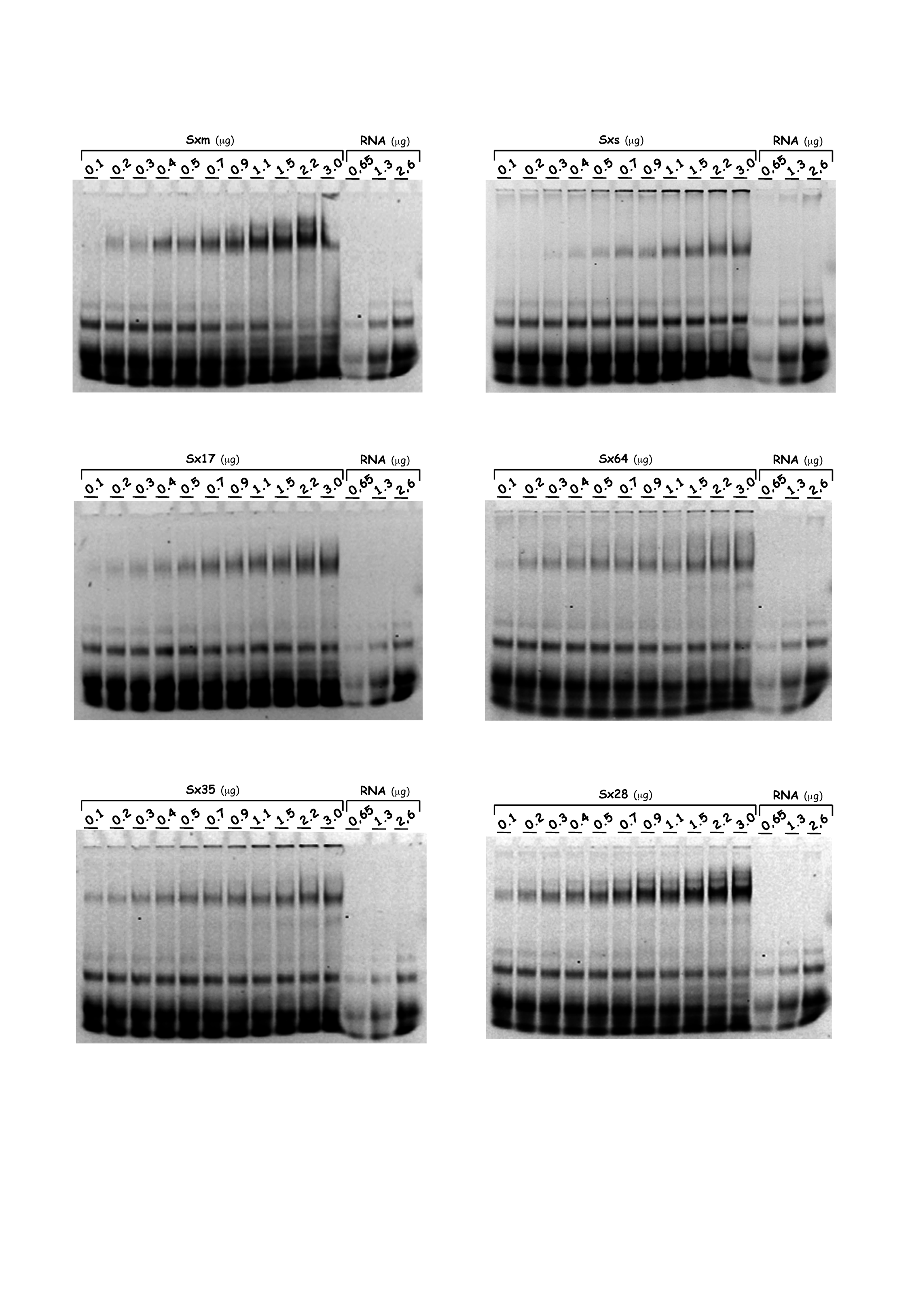

Supplement: Figure S1 — Examples of binding of SXL proteins to one single SXL-binding site. The amount of SXL proteins and RNA substrate is indicated in µg above each lane. (TIFF) [file pone.0065171.s001.tiff]

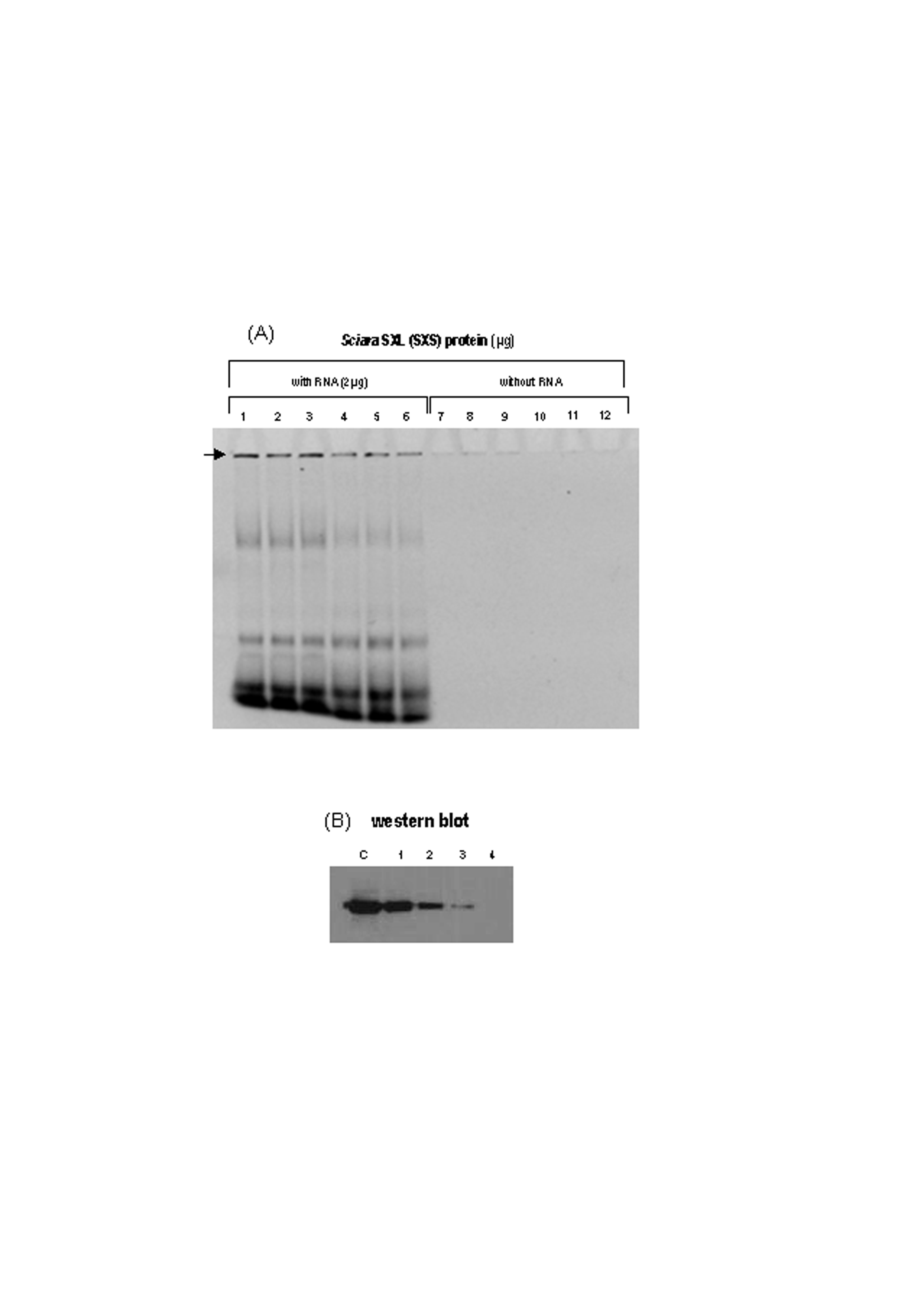

Supplement: Figure S2 — EMSA (A) and Western-blot (B) for the interaction between the Sciara SXL (SXS) protein and the Drosophila SXL-binding site. (A) The RNA sequence is described in Materials and Methods. Lanes 1, 2, 3, 7, 8 and 9 corresponded to 3 µg of SXS protein used in the reaction, whereas lanes 4, 5, 6, 10, 11 and 12 corresponded to 0,7 µg of SXS protein used in the reaction. The arrow in indicates the wells of the gel. (B) Western-blot to demonstrate to existence of SXS protein retained in the wells of the EMSA shown in (A). The material retained in the wells was extracted and used for the Western–blot. Lane 1 corresponds to the material retained in the wells of lanes 1, 2 and 3; lane 2 corresponds to the material retained in the wells of lanes 7, 8 and 9; lane 3 corresponds to the material retained in the wells of lanes 4, 5 and 6, and lane 4 corresponds to the material retained in the wells of lanes 10, 11 and 12. C stands for the SXS protein alone and used as control. The Western-blot was hybridised with the serum against the Sciara SXL protein [36]. (TIFF) [file pone.0065171.s002.tiff]

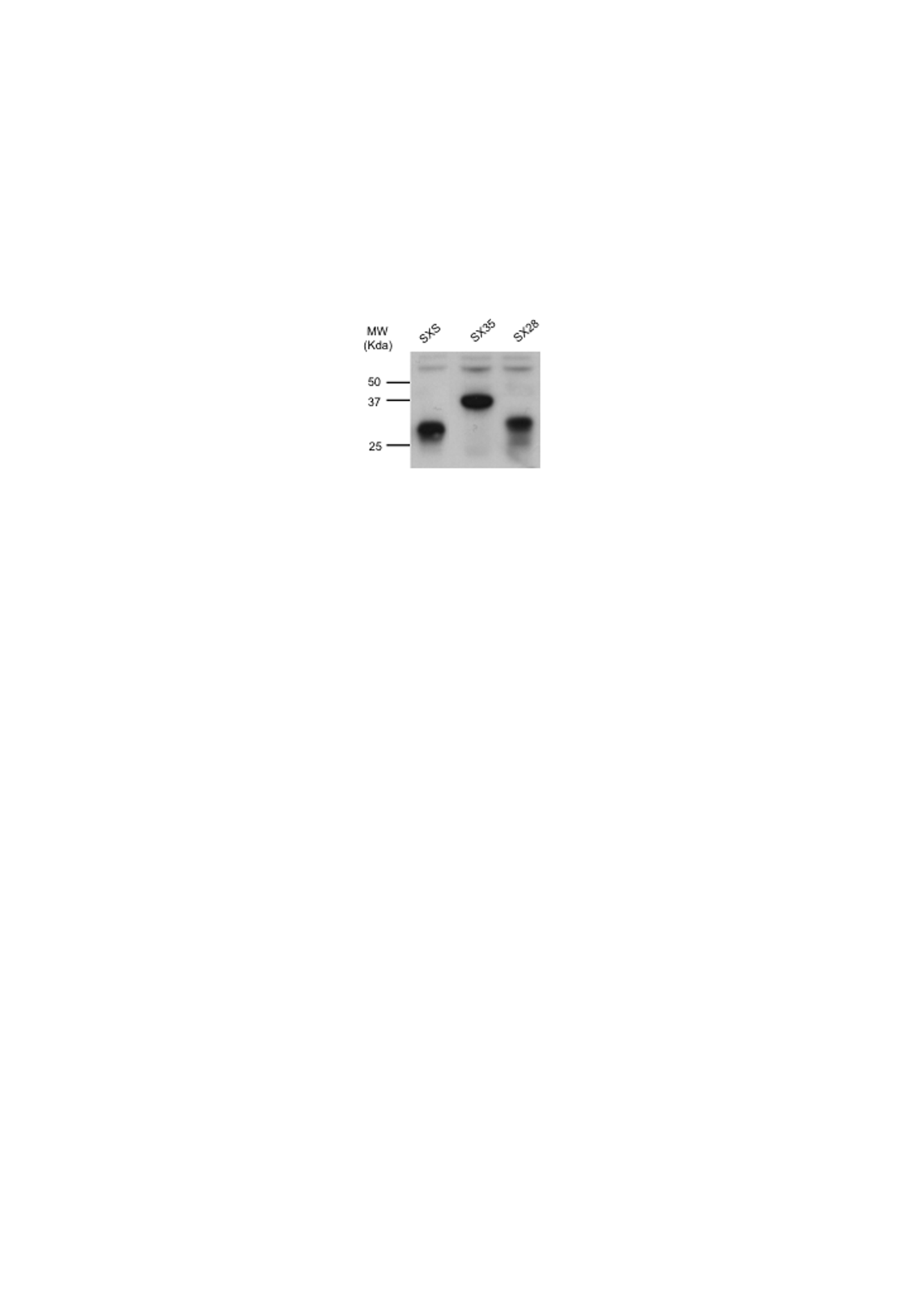

Supplement: Figure S3 — Western-blot hybridised with serum against the Sciara SXL protein [36] showing the expression of the transgenic SXL proteins. The antibody does not recognise Drosophila SXL protein [36]. See text for details. (TIFF) [file pone.0065171.s003.tiff]

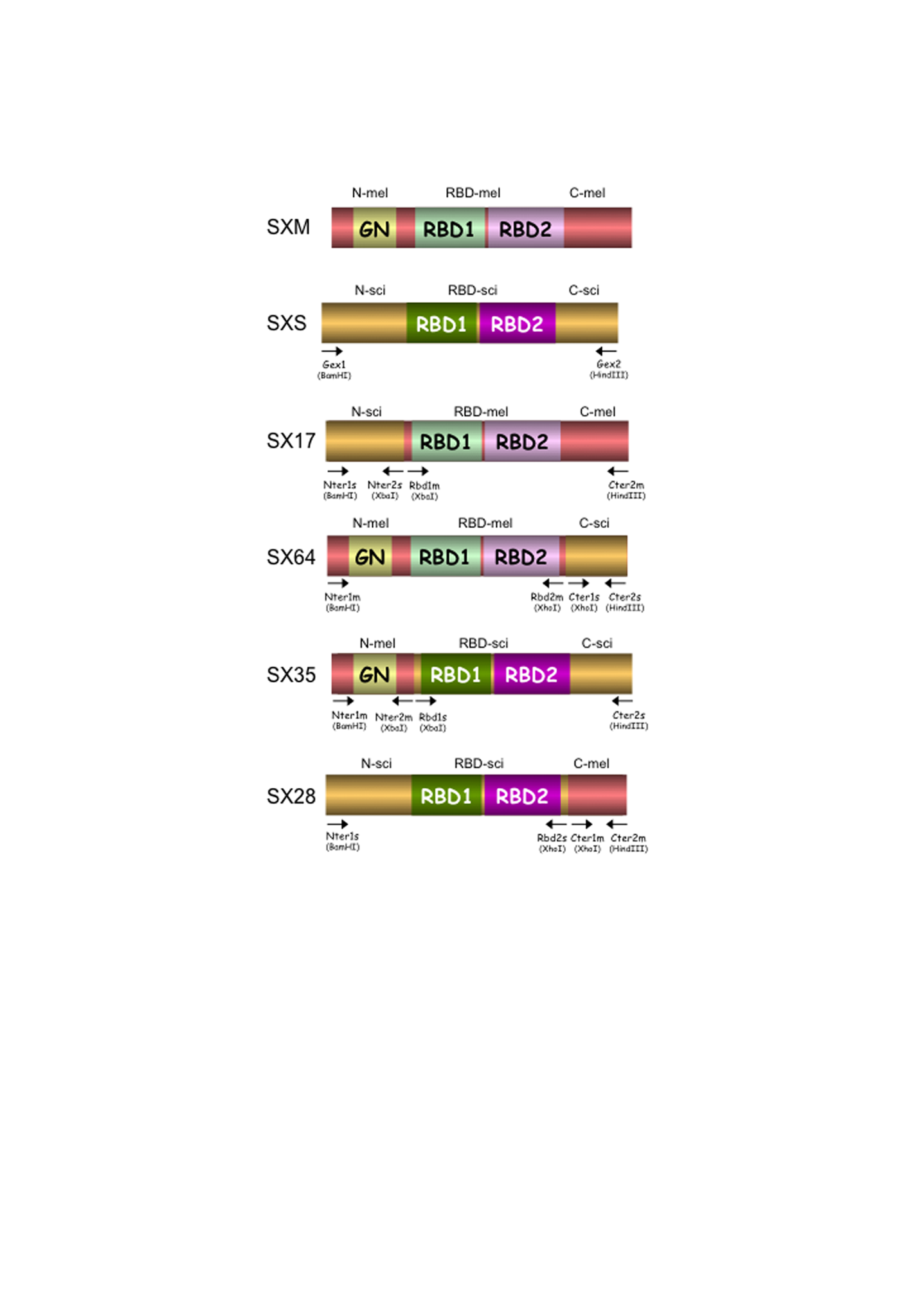

Supplement: Figure S4 — Scheme showing the Drosophila-Sciara chimeric SXL proteins, where the location of the primers used for their construction is indicated. The sequences of the primers and the added sequences for the restriction enzymes are described in Materials and Methods. N-mel, RBD-mel and C-mel stand, respectively, for the amino-terminal domain, the two RNA-binding domains and the carboxyl-terminal domain of Drosophila SXL. N-sci, RBD-sci and C-sci stand, respectively, for the amino-terminal domain, the two RNA-binding domains and the carboxyl-terminal domain of Sciara SXL. (TIFF) [file pone.0065171.s004.tiff]
